# Biomarkers in the molecular pathogenesis of esophageal (Barrett) adenocarcinoma

**DOI:** 10.3747/co.v13i1.76

**Published:** 2006-02

**Authors:** L.J. Williams, D.L. Guernsey, A.G. Casson

**Affiliations:** * Department of Surgery (Division of Thoracic Surgery), Dalhousie University and the QEII Health Sciences Centre, Halifax, Nova Scotia; † Department of Pathology (Division of Molecular Pathology and Molecular Genetics), Dalhousie University and the QEII Health Sciences Centre, Halifax, Nova Scotia

**Keywords:** Barrett esophagus, esophageal adenocarcinoma, molecular pathogenesis, biomarkers

## Abstract

Since the early 1970s, a dramatic change has occurred in the epidemiology of esophageal malignancy in both North America and Europe: the incidence of adenocarcinomas of the lower esophagus and esophagogastric junction is increasing. Several lifestyle factors are implicated in this change, including gastroesophageal reflux disease (gerd). Primary esophageal adenocarcinomas are thought to arise from Barrett esophagus, an acquired condition in which the normal esophageal squamous epithelium is replaced by a specialized metaplastic columnar-cell-lined epithelium.

Today, gerd is recognized as an important risk factor in Barrett esophagus. Progression of Barrett esophagus to invasive adenocarcinoma is reflected histologically by the metaplasia–dysplasia–carcinoma sequence. Although several molecular alterations associated with progression of Barrett esophagus to invasive adenocarcinoma have been identified, relatively few will ultimately have clinical application. Currently, the histologic finding of high-grade dysplasia remains the most reliable predictor of progression to invasive esophageal adenocarcinoma. However other promising molecular biomarkers include aneuploidy; 17p loss of heterozygosity, which implicates the *TP53* tumour suppressor gene; cyclin D1 protein overexpression; and p16 alterations. It is anticipated that models incorporating combinations of objective scores of sociodemographic and lifestyle risk factors (that is, age, sex, body mass index), severity of gerd, endoscopic and histologic findings, and a panel of biomarkers will be developed to better identify patients with Barrett esophagus at increased risk for malignant progression, leading to more rational endoscopic surveillance and screening programs.

## 1. INTRODUCTION

Since the early 1970s, a dramatic change has occurred in the epidemiology of esophageal malignancy in both North America and Europe: the incidence of adenocarcinomas of the lower esophagus and esophagogastric junction (egj) is increasing, with stability in the incidence of squamous cell carcinoma 1–3. The reasons for this change are unclear and controversial, but several lifestyle factors have been proposed 4–7. Gastroesophageal reflux disease (gerd) has been demonstrated to have a strong statistical association with risk of esophageal adenocarcinoma and its premalignant lesion, Barrett esophagus 8–10. Also, prevalence rates for obesity in the general population have increased markedly since the 1970s 11–13, and a number of case-control studies support the role of obesity as a major risk factor for esophageal adenocarcinoma 14–19. However, the precise biologic mechanisms underlying the reported associations between body mass, gerd, and esophageal adenocarcinoma have yet to be defined. Although diet 11,16,20–22 and tobacco and alcohol consumption 15,16,23,24 are well-established risk factors for squamous cell carcinoma of the esophagus, their contribution to the pathogenesis of esophageal adenocarcinoma remains unclear.

Primary esophageal adenocarcinomas are thought to arise from Barrett esophagus, an acquired condition in which the normal esophageal squamous epithelium is replaced by a specialized metaplastic intestinal columnar-cell-lined epithelium 25,26. Although the definition of Barrett esophagus (Barrett epithelium, Barrett mucosa) has been somewhat variable in the past, the American College of Gastroenterology currently defines Barrett esophagus as “a change in the esophageal epithelium of any length that can be recognized at endoscopy and is confirmed to have intestinal metaplasia by biopsy” 27,28. Progression of Barrett esophagus to invasive adenocarcinoma is reflected histologically by the metaplasia–dysplasia–carcinoma sequence 29.

An established risk factor for Barrett esophagus is gerd, and consequently, a plausible link exists between gerd, Barrett esophagus, and esophageal adenocarcinoma 30. The hypothesis is that gerd results in acute mucosal injury (esophagitis), thereby promoting cellular proliferation and inducing specialized columnar metaplasia of the normal squamous epithelium lining the esophagus. Dysplasia is widely regarded as the precursor of invasive cancer, and high-grade dysplasia in Barrett epithelium is frequently associated with esophageal adenocarcinoma. That association underlies the rationale for endoscopic surveillance programs: to detect malignancy at an earlier and potentially curable stage. However, the surveillance strategy remains controversial.

The recent identification of molecular markers associated with the Barrett metaplasia–dysplasia–carcinoma progression may have potential clinical application to identify individuals with Barrett esophagus who are at increased risk for progression to invasive adenocarcinoma 29,31–33.

The present review focuses on the pathogenesis of Barrett esophagus and esophageal adenocarcinoma, with emphasis on selected molecular biomarkers that may have future clinical application to the management of this disease.

## 2. PATHOLOGY

### 2.1 Intestinal Metaplasia

The diagnosis of intestinal metaplasia is made histologically and is characterized by the presence of goblet cells 34,35. Alcian blue may complement the histologic diagnosis, because mucin produced by goblet cells stains an intense blue colour. Less intense staining is also seen with some reactive conditions and should not be confused with specialized intestinal metaplasia unless accompanied by the distinctive goblet cell morphology.

The definition of intestinal metaplasia (Barrett esophagus) may be further categorized by endoscopic measurement of the length of the columnar epithelium. Classic “long-segment” Barrett esophagus refers to specialized intestinal metaplasia extending more than 3 cm above the anatomic egj 36,37; “short-segment” Barrett esophagus is defined as intestinal metaplasia within 3 cm of the egj 38,39. When no obvious columnar epithelium is seen endoscopically, microscopic foci of specialized intestinal metaplasia may be identified at biopsy 40,41. This latter entity has been called either “cardia intestinal metaplasia” or “ultra-short segment” Barrett esophagus, and its significance is extremely controversial 39.

From a practical standpoint, intestinal metaplasia of the esophagus and cardia cannot be accurately differentiated on the basis of routine histology alone. Recent immunohistochemical studies have reported a potential role for cytokeratins 7 (CK7) and 20 (CK20) in differentiating the two 42. In esophageal intestinal metaplasia, CK7 positivity is found in superficial and deep glands, and CK20 positivity is limited to superficial glands (“Barrett CK 7/20 pattern”). In cardia intestinal metaplasia, CK7 immunoreactivity is absent (or weak or patchy), but CK20 positivity is seen in both superficial and deep glands. Although early reports suggest that CK 7/20 immunostaining patterns are highly sensitive and specific, routine application of these immunohistochemical techniques requires further critical evaluation. Interpretation of CK immunoreactivity should be applied in conjunction with current clinical, endoscopic, and histologic findings.

The reported prevalence of Barrett esophagus has varied between populations, and most studies have reported incidence and prevalence rates for the classic long-segment disease 43. However, several trends are apparent, with male sex and increasing age being associated with an increased prevalence of Barrett esophagus 44,45. For patients who undergo endoscopy for upper gastrointestinal symptoms (predominantly reflux-related), current estimates suggest a prevalence for Barrett esophagus of about 3%–8%; this compares with a reported prevalence of about 1% in patients who undergo endoscopy for any clinical indication 46–48. However, autopsy series suggest a much higher prevalence (approximately 20 times higher) in the general population 49. Currently, it is unclear whether the prevalence of Barrett esophagus is increasing 50,51, but preliminary data from the United Kingdom (adjusted for increasing numbers of endoscopic procedures) suggests a real increase in prevalence 52. Other studies have reported a stable prevalence for long-segment disease, suggesting that the prevalence of short-segment Barrett esophagus is what has increased—likely as a result of increased awareness of the condition 53. Estimates of the prevalence of short-segment Barrett in unselected patients currently range from 2% to 13%, and estimates for cardia intestinal metaplasia of up to 35% are reported 38,40,41,48,54.

### 2.2 Dysplasia

Dysplasia within a segment of Barrett esophagus can be identified histologically based on phenotypic nuclear alterations resulting from dna abnormalities. Such dysplasia is generally classified as indefinite, low-grade, or high-grade 28,35,55. Low-grade and high-grade dysplasia may be differentiated based on nuclear localization in relation to the luminal surface of the cell. Unfortunately, the histologic diagnosis of dysplasia is largely subjective, and it is generally accepted that considerable intra- and inter-observer variation occurs. Interpretation of dysplasia may be further complicated when atypical epithelial cells, arising from a background of active inflammation, are present.

Although the natural history of dysplasia is not known, it was recently reported that the presence of low-grade dysplasia in Barrett epithelia conferred an increased risk of progression to malignancy 56. Currently, however, high-grade dysplasia (hgd) is regarded as the most reliable predictor for progression to invasive cancer; hgd is frequently associated with esophageal adenocarcinoma 41,55,57. Unfortunately, the rate of progression of hgd to invasive adenocarcinoma is quite variable; reports have indicated that most patients with hgd actually die from non-malignant causes 58,59.

Relatively few studies have investigated the prevalence of dysplasia within Barrett epithelia. Based on careful pathologic examination of resected esophageal adenocarcinomas, dysplastic change is reported in a relatively high percentage of associated Barrett epithelia 34,35,55,57. However, the prevalence of dysplasia in patients with Barrett esophagus who undergo endoscopy for any reason is estimated to be below 10% 44,54,60,61.

### 2.3 Adenocarcinomas of the Esophagus and Cardia

The definition of primary esophageal adenocarcinoma is controversial, especially when the tumour involves the egj or cardia. The Siewert system is increasingly being used to classify adenocarcinomas of the esophagus and esophagogastric junction. This system is based on an estimate of the tumour centre in relation to the egj^62^. Type i (esophageal adenocarcinomas) arise 1 cm to 5 cm above the egj, and type ii (cardia adenocarcinomas) arise from the region 1 cm above to 2 cm below the egj. Type iii (subcardia gastric adenocarcinomas) arise 2 cm to 5 cm below the egj. One disadvantage of the Siewert classification is that precise measurements may be difficult for large, advanced-stage tumours.

Since the mid-1990s, our group has used strict clinicopathologic criteria to stratify primary esophageal adenocarcinomas (type i) from adenocarcinomas arising at the egj (type ii) 63. Based on clinical, endoscopic, radiologic, operative, and pathologic findings, we have defined primary esophageal adenocarcinomas as follows:

1. Presence of an associated Barrett epithelium
2. A tumour mass that involves more than 75% of the tubular esophagus
3. Histologic evidence of direct invasion of peri-esophageal tissues
4. Minimal gastric involvement
5. Clinical symptoms of esophageal obstruction (that is, dysphagia)

The most important criterion for establishing a diagnosis of a primary esophageal adenocarcinoma is the presence of Barrett epithelium. However, because Barrett epithelium may not be identified in up to 50% of surgically resected esophageal adenocarcinomas (likely as a consequence of tumour progression), criteria 2 to 5 should be met to establish a primary esophageal origin (as opposed to a cardia, subcardia, or gastric origin).

The prevalence of esophageal adenocarcinoma has recently been well studied and, as mentioned earlier, has increased steadily since the mid-1970s. Current estimates of the annual incidence of adenocarcinoma of the esophagus and egj range from 0.74 to 1.34 per 100,000 population 43. Older series suggested an annual risk of adenocarcinoma in Barrett esophagus of 1%–2%, but more recent studies suggest that the risk of adenocarcinoma is approximately 0.4% per person–year of follow-up in patients with Barrett esophagus as compared with 0.07% per person–year in patients without Barrett esophagus 64.

## 3. ENDOSCOPIC SURVEILLANCE AND BARRETT ESOPHAGUS

The goal of any cancer surveillance program is the detection of premalignant or early invasive disease which, when treated, will ultimately result in improved survival. For patients with Barrett esophagus, endoscopic surveillance refers to esophagogastroscopy and biopsy performed at regular intervals to detect hgd or cancer at an early and potentially curable stage.

Table I summarizes the current guidelines for endoscopic surveillance from the American College of Gastroenterology 65. Recommendations are based on the highest grade of dysplasia identified by histology at baseline and confirmed by two expert gastrointestinal pathologists. At each surveillance interval, a standard protocol of four quadrant biopsies for every 2 cm length of Barrett epithelium (in the absence of visible abnormalities) is currently advised 28.

The efficacy and utility of Barrett surveillance is controversial. Among the proposed disadvantages are the difficulty of identifying early neoplastic lesions with current endoscopic techniques and the frequency of sampling error 66. Furthermore, even among expert histopathologists, substantial intra- and inter-observer variability in grading dysplasia is seen, making accurate diagnosis difficult. Surveillance endoscopy can be expensive and time-consuming, and given the low absolute incidence of esophageal adenocarcinoma among patients with Barrett esophagus, the cost-effectiveness of such surveillance has been questioned 67,68. Additionally, the ability of surveillance to detect earlier cancers and to improve outcome is unclear. Retrospective data suggest that esophageal adenocarcinomas detected during a surveillance program are more likely to have an earlier stage and improved 2-year and 5-year survival as compared with those detected in patients not involved in a surveillance program 69–71.

It has been suggested that new strategies are required to improve the efficacy of surveillance for Barrett esophagus. Proposed methods of reducing endoscopic variability and sampling error include the use of chromoendoscopy, magnification endoscopy, fluorescent endoscopy, or optical coherence tomography 66. Other proposed approaches include the use of alternative techniques for tissue sampling (such as brush cytology or mucosal stripping), combined with the evaluation of molecular markers to help identify patients at high risk for progression to invasive esophageal adenocarcinoma.

### 3.1 Pathogenesis

Although the precise causes and natural history of Barrett esophagus remain unknown, the condition is generally accepted to be acquired as a result of gerd. This theory is supported by a number of physiologic abnormalities that have been identified in patients with Barrett esophagus—among them, increased acid exposure, a defective lower esophageal sphincter, and impaired esophageal motility and clearance 37. Furthermore, a role for the reflux of duodenal contents in the pathogenesis of esophagitis and Barrett esophagus has been proposed. Pure alkaline reflux is thought to be rare, but it has been suggested that a mixed refluxate consisting of acid, bile, lysolecithin, and pancreatic enzymes may cause more esophageal mucosal damage than acid alone 72,73. Bile acids are thought to alter the ionic permeability of mucous membranes, with back-diffusion of hydrogen ions and resultant intracellular acidification 74.

The hypothesis is that gerd leads to acute mucosal injury, promotes cellular proliferation, and induces specialized intestinal metaplasia of the esophagus. Studies have shown Barrett esophagus to be hyperproliferative, reflected by an increased S-phase fraction on flow cytometry 75 and by immunohistologic detection of proliferating cell nuclear antigen (pcna) 76,77 and of a cell nuclear proliferation–associated antigen (Ki67) 78. Specifically, pcna immunostaining is limited to the basal layer of metaplastic Barrett epithelia; in hgd, it extends to the more superficial layers. Similar patterns of staining for Ki67 suggest a functional instability of Barrett mucosa.

To date, no genetic locus for familial gerd or Barrett esophagus (or both) has been reported 79. Associations have been reported between Barrett esophagus and various intrinsic esophageal diseases including scleroderma 80 and esophageal conditions subsequent to gastrectomy 81, lye ingestion 82, and myotomy for achalasia 83. Use of anticancer chemotherapy has also been associated with Barrett esophagus 84. However, these diseases and conditions frequently have associated abnormalities at the egj, predisposing to gerd or to stasis.

Similarly, the causes and pathogenesis of cardia intestinal metaplasia are unknown. A number of reports suggest that this entity may represent an early manifestation of gerd (and hence be associated with Barrett esophagus); others have found stronger associations with chronic gastritis, *Helicobacter pylori* infection, gastric intestinal metaplasia, and gastric malignancy 85–87. Of particular interest is the association with *H. pylori,* because gastric infection with *S100A8-*positive strains (formerly called *CAGA-*positive strains) has been found to have an inverse association with the development of esophageal adenocarcinoma 88. Currently, it has been suggested that cardia intestinal metaplasia be considered a separate entity until its causes, pathogenesis, and association with malignancy are more clearly defined.

### 3.2 Candidate Biomarkers for Esophageal (Barrett) Adenocarcinoma

**Figure 1:**
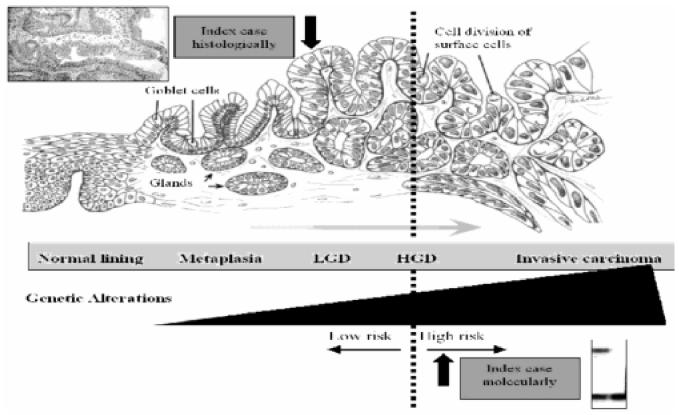
Barrett metaplasia–dysplasia–carcinoma sequence. Histologically, the index case is at low risk for malignant progression, but the accumulation of molecular alterations may confer an increased risk for progression to invasive esophageal adenocarcinoma. lgd = low-grade dysplasia; hgd = high-grade dysplasia. (Reprinted with permission from Wiley–Liss, Inc., a subsidiary of John Wiley & Sons, Inc.)

A number of molecular alterations have been reported in Barrett esophagus, and these molecular alterations are implicated in the molecular pathogenesis of esophageal adenocarcinoma 29,31–33. Figure 1 outlines the accumulation of these molecular alterations in the metaplasia–dysplasia–carcinoma sequence. Molecular “biomarkers” may have potential clinical application in these areas:

- Molecular diagnosis for early detection of hgd or invasive adenocarcinoma
- Prediction of risk for disease progression in endoscopic surveillance programs
- Staging and prognosis
- Prediction of chemosensitivity
- Intermediate biomarkers in chemoprevention studies
- Novel targets for anticancer therapies

Only a limited number of molecular biomarkers are anticipated to ultimately have clinical application. The introduction of selected biomarkers into clinical practice will require careful evaluation. To facilitate the process of introducing advances in basic science into clinical practice, the National Cancer Institute (nci) Early Detection Research Network (edrn) has developed five phases to validate novel biomarkers used in the early detection of cancer (see Table II 89) Notably, however, not all biomarkers need to progress sequentially through each of the phases before recommendations regarding their clinical application can be made. Rather, the recommendations were developed as a conceptual framework to help coordinate biomarker research.

To date, no phase 5 studies have been conducted to evaluate potential biomarkers associated with Barrett esophagus. By contrast, several biomarkers have been identified and evaluated in phase 1 and 2 studies, and these markers have been reviewed in detail elsewhere 29,31–33.

The discussion that follows therefore focuses on the recent results of a limited number of phase 3 and 4 studies that have evaluated selected biomarkers with potential clinical application in the management of Barrett esophagus and esophageal adenocarcinoma.

#### 3.2.1 Ploidy

Several studies have reported that aneuploidy (or abnormal cell nuclear dna content) in Barrett epithelia is associated with risk of progression to malignancy 75,90–93. Furthermore, these phase 1–3 studies suggest that the prevalence of aneuploidy increases with the degree of dysplasia, as determined by tissue histology.

A recently published and ongoing phase 4 study, which has used a well-established endoscopic biopsy protocol to prospectively evaluate more than 300 patients for 15 years, has provided further convincing evidence for using flow cytometry to determine tissue ploidy in Barrett epithelia 94–99.

In brief, patients with Barrett esophagus who had no, indefinite, or low-grade dysplasia at baseline biopsy and a diploid cell population by flow cytometry (that is, no aneuploidy or increased 4N fraction) appeared to be at low risk for progression to adenocarcinoma 95. It was suggested that this group of patients could undergo endoscopic surveillance at intervals of up to 5 years. Patients whose baseline biopsies had aneuploidy, tetraploidy (4N), or hgd had 5-year cancer incidences of 43%, 56%, and 59% respectively, prompting a recommendation for more frequent surveillance for that group. Interestingly, patients who showed no aneuploidy or tetraploidy by flow cytometry, but who progressed to invasive esophageal adenocarcinoma, all had hgd at baseline biopsy 95. Subsequent analysis of study data has determined specific ploidy variables that appear to be even more highly predictive of cancer progression—namely, aneuploid content greater than 2.7N, and a 4N fraction greater than 6% 97. For esophageal adenocarcinoma, tumour ploidy has been reported to be associated with advanced-stage disease, lymph node metastasis, and reduced survival; but overall, these findings have been inconsistent 98–100.

Flow cytometry has also been used to study cell-cycle kinetics, including S-phase fraction, in esophageal pre-malignancy in a number of phase 1 and 2 studies 75,91,97,101. In the only substantive phase 4 study of Barrett esophagus performed to date, the S-phase fraction was shown by univariate analysis to be a predictor of cancer risk, but not a significant independent risk factor in a multivariate model incorporating ploidy and dysplasia 97.

#### *3.2.2* TP53 *Tumour Suppressor Gene*

The tumour suppressor gene *TP53* is located on chromosome 17p13 and encodes a 53-kDa polypeptide (Tp53) that regulates cell cycle progression, dna repair, apoptosis, and neovascularization in both normal and malignant cells via highly complex dna and protein interactions 102,103. By inducing expression of *CDKN1A* (also called *P21, WAF1*), which sequesters a number of cyclin-dependent kinases (cdks), Tp53 mediates both G1 and G2/M arrest. Point mutations leading to loss of function of *TP53* is a common mechanism of inactivation, and more than 90% of *TP53* mutations have been located in the conserved dna binding domain (exons 5–8). Through the 1990s, *TP53* was extensively characterized; it appears to have a central role in human malignancy 104,105.

Mutations in the *TP53* gene were initially reported in primary esophageal adenocarcinomas and associated Barrett epithelium in 1991 106. These findings were subsequently confirmed in several phase 1 and 2 studies, and the spectrum of *TP53* alterations in Barrett esophagus has been extensively characterized 107. The finding of *TP53* mutations in non-dysplastic Barrett epithelia suggests that *TP53* may be altered early in the metaplasia–dysplasia–carcinoma sequence, and it may therefore be a useful biomarker in endoscopic surveillance programs.

In a 10-year prospective study of surgically resected esophageal adenocarcinomas, *TP53* mutations were associated with poor tumour differentiation and with reduced disease-free and overall survival following surgical resection 108. Of particular biologic interest was the observation that patterns of *TP53* mutation in esophageal adenocarcinomas were predominantly G:C to A:T transitions at CpG dinucleotides, suggesting that *TP53* mutations result from endogenous mechanisms that likely involve spontaneous deamination into thymine of the 5′-methylated cytosine that frequently occurs at CpG di-nucleotides. Because this mechanism is enhanced by exposure to oxy-radicals and nitro-radicals, we hypothesized that local overproduction of nitric oxide, a consequence of chronic gerd, may enhance the rate of formation of spontaneous *TP53* mutations in Barrett esophagus.

Although no phase 4 studies have evaluated *TP53* mutations or protein overexpression in Barrett epithelia as predictors of malignant progression, loss of heterozygosity (loh) of 17p (inclusive of *TP53*) was evaluated in one ongoing phase 4 study in conjunction with flow cytometry 96. The prevalence of 17p loh ranged from 6% in non-dysplastic Barrett epithelia to 57% in hgd and was a significant independent predictor of progression to esophageal adenocarcinoma, with a relative risk of 16. In this study of 325 patients with Barrett esophagus, only 6 of 26 patients who progressed to malignancy lacked 17p loh. And 17p loh was also associated with increased risk for aneuploidy, tetraploidy, and hgd, with relative risks of 7.5, 6.1, and 3.6 respectively.

#### 3.2.3 Cyclin D1

Cyclin D1 is a key regulator of cell-cycle progression, particularly at the transition from G1 to the S phase; it is encoded by the *CCND1* gene located on chromosome 11q13 109. Several phase 1 and 2 studies have implicated cyclin D1 in esophageal malignancy, and overexpression of cyclin D1 protein has been reported in up to 64% of adenocarcinomas and associated Barrett epithelia 110,111. Recently, a phase 3 case-control study reported that immunohistochemical overexpression of cyclin D1 in patients with Barrett esophagus was associated with an increased risk for progression to esophageal adenocarcinoma 111.

As a result of a single base polymorphism (G870A) of *CCND1,* alternative gene splicing is thought to give rise to two functional transcripts 112–114. The normal gene transcript (cyclin D1a) interacts with, and activates the G1 cdks 4 and 6 (CDK4/6); the resulting complex phosphorylates the *RB1* tumour suppressor gene, thereby resulting in cell-cycle progression to S phase. The variant transcript (cyclin D1b), a consequence of the polymorphic A-allele, encodes a truncated protein isoform with an altered C-terminal domain that has been implicated in neoplastic transformation 113,114. A report from a prospective case-control (phase 4) study said that individuals with the *CCND1* A/A genotype were at increased risk for gerd, Barrett esophagus, and esophageal adenocarcinoma, supporting the hypothesis that this polymorphism is an individual susceptibility factor in the molecular progression of esophageal adenocarcinoma 115.

#### *3.2.4* CDKN2A *Gene*

The *CDKN2A* gene (formerly called *p16^INK4a^*), which is localized to chromosome 9p21, encodes a protein, P16, that belongs to a family of cdk inhibitors. The P16 protein binds to and inhibits CDK4/6, resulting in reduced phosphorylation of *RB1* and inhibition of cell-cycle progression through G1. An alternative transcript (formerly called *p14^ARF^*) functions to sequester *MDM2,* thereby stabilizing the *TP53* tumour suppressor gene 116.

Alterations of *CDKN2A* are reported frequently in various human malignancies, but mechanisms of *CDKN2A* inactivation appear to vary between tumour types. Point mutations in Barrett esophagus and esophageal adenocarcinoma are relatively uncommon, but 9p loh and promoter hypermethylation appear to be frequent mechanisms of *CDKN2A* inactivation 117,118. Although *CDKN2A* alterations have been the subject of phase 1 and 2 studies only, they are increasingly recognized as critical early molecular lesions associated with clonal proliferation within Barrett epithelia 116.

## 4. SUMMARY

Despite advances in multimodality therapy, esophageal (Barrett) adenocarcinoma remains a highly lethal malignancy. To substantially improve outcomes with this disease, future management strategies will need to focus on prevention and early detection based on an improved understanding of esophageal tumour biology. Although several molecular alterations in the progression of Barrett esophagus to invasive esophageal adenocarcinoma have been identified, relatively few will ultimately prove to have clinical application. Currently, hgd remains the most reliable predictor of progression to invasive esophageal adenocarcinoma, but potentially promising biomarkers include aneuploidy (dna content greater than 2.7N, or 4N fraction greater than 6%, or both) 95,97, 17p loh and *TP53* mutations 96, cyclin D1 protein overexpression 111, and *CDKN2A* alterations 116,118. It is anticipated that models incorporating a combination of objective scores of sociodemographic and lifestyle risk factors (that is, age, sex, body mass index), severity of gerd, endoscopic and histologic findings, and a panel of biomarkers will be developed to better identify patients with Barrett esophagus at increased risk for malignant progression, leading to more rational endoscopic surveillance and screening programs.

## Figures and Tables

**Table I:** American College of Gastroenterology guidelines for Barrett esophagus surveillance 65

*Dysplasia*	*Management*	*Follow-up intervention*
None	Endoscopy and biopsy showing Barrett-related columnar metaplasia Treat esophagitis, if present, until healed Repeat endoscopy in 1–3 months and obtain four-quadrant biopsies every 2 cm to establish baseline diagnosis	Endoscopy up to 3 years
Low grade	As above Repeat endoscopy with biopsy shows low-grade dysplasia	Endoscopy annually until no dysplasia
High grade	As above	Surgical resection
	Repeat endoscopy with four-quadrant biopsies every 1 cm to exclude cancer and document high-grade dysplasia Confirm with two expert pathologists	Endoscopy every 3 months (if medically high risk) Endoscopic mucosal resection or ablation

**Table II:** Phases of biomarker development for early detection of cancer^89^

Phase 1	Preclinical exploratory	Single laboratory identifies a promising gene Comparison of tumour and normal tissues Selected tissue samples, often late stage Limited outcomes/predictive value meaningless
Phase 2	Clinical assay and validation	Phase 1 findings confirmed by other laboratories Reproducibility and development of laboratory assays
Phase 3	Retrospective longitudinal	Wider spectrum of tissues studies (tissue bank) Biomarkers evaluated in premalignant disease (assessment of “lead time”) Criteria for a positive screening test defined Limited outcomes
Phase 4	Prospective screening	Prospective assessments of outcomes: positive predictive tests Feasibility for future controlled trials (phase 5) Ethical issues
Phase 5	Cancer control	Randomized trial of screening vs. “usual care” Assessment of reduced cancer mortality by screening Compliance, acceptability Cost–benefit analysis
